# Design of a Zn Single-Site Curing Activator for a More Sustainable Sulfur Cross-Link Formation in Rubber

**DOI:** 10.1021/acs.iecr.1c01580

**Published:** 2021-07-07

**Authors:** Silvia Mostoni, Massimiliano D’Arienzo, Barbara Di Credico, Lidia Armelao, Marzio Rancan, Sandra Dirè, Emanuela Callone, Raffaella Donetti, Antonio Susanna, Roberto Scotti

**Affiliations:** † Department of Materials Science, INSTM, University of Milano-Bicocca, Via R. Cozzi 55, Milano 20125, Italy; ‡ Institute of Condensed Matter Chemistry and Technologies for Energy, National Research Council of Italy, ICMATE-CNR, via Marzolo 1, Padua 35131, Italy; § Department of Chemical Sciences, University of Padua, Via Marzolo 1, Padua 35131, Italy; ∥ Department of Chemical Sciences and Materials Technologies, National Research Council of Italy, DSCTM-CNR, Piazzale A. Moro 7, Rome 00185, Italy; ⊥ “Klaus Müller” Magnetic Resonance Lab., Dept. of Industrial Engineering, University of Trento, Via Sommarive 9, Trento 38123, Italy; # Pirelli Tyre SpA, Viale Sarca 222, Milano 20126, Italy

## Abstract

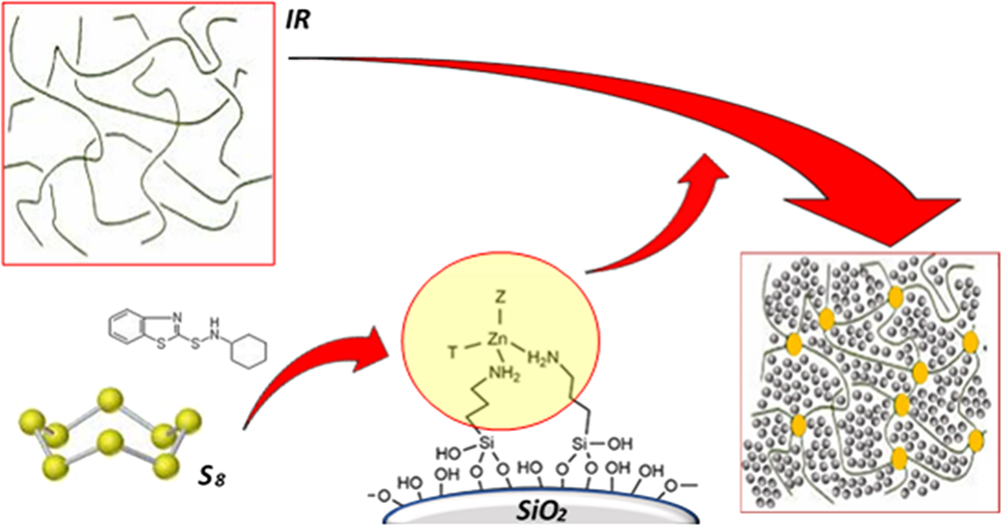

ZnO is a worldwide used activator for a rubber vulcanization process, which promotes fast curing kinetics and high cross-linking densities of rubber nanocomposites (NCs). However, its extended use together with leaching phenomena occurring during the production and life cycle of rubber products, especially tires, entails potential environmental risks, as ecotoxicity toward aquatic organisms. Pushed by this issue, a novel activator was developed, which introduces highly dispersed and active zinc species in the vulcanization process, reducing the amount of employed ZnO and keeping high the curing efficiency. The activator is constituted by Zn(II) single sites, anchored on the surface of SiO_2_ nanoparticles (NPs) through the coordination with functionalizing amino silane groups. It behaves as a double-function material, acting at the same time as a rubber reinforcing filler and a curing activator. The higher availability and reactivity of the single-site Zn(II) centers toward curative agents impart faster kinetics and higher efficiency to the vulcanization process of silica/isoprene NCs, compared to conventionally used ZnO activators. Moreover, the NCs show a high cross-linking degree and improved dynamic mechanical properties, despite the remarkably lower amount of zinc employed than that normally used for rubber composites in tires. Finally, the structural stability of Zn(II) single sites during the curing reactions and in the final materials may represent a turning point toward the elimination of zinc leaching phenomena.

## 1 Introduction

Rubber is a material used in many widespread applications (e.g., tires, tubes, footwear and gloves, glues, etc.),^1−4^ thanks to its unique properties, as low hardness, high elasticity, and high elongation at break. For most of the applications, rubber undergoes a curing process to convert the raw sticky polymer into an elastic material by cross-linking the rubber chains. In addition, its mechanical properties are commonly enhanced by adding reinforcing nanofillers, e.g., SiO_2_, silicates, POSS, and carbon black^5−9^ to the rubber matrix, which promote the formation of a percolative filler network inside the rubber nanocomposites (NCs).^10−12^ Among curing methods, vulcanization is a consolidated key technology in the rubber industry to cure unsaturated rubber chains through sulfur cross-links at high temperature and formation of poly- to monosulfide bridges between them.^13,14^

Despite the technological level of rubber materials and in particular of tires, which represent worldwide their major application, in the last decades, the sustainability of their production, use, and disposal has become a main issue.^15,16^ Nowadays, in the frame of a gradual but still far transformation of the rubber materials’ life cycle into a circular economy model, several efforts are required to promote a more sustainable use/reuse of the raw materials in rubber products, lowering the use of toxic and potentially harmful materials, at the same time keeping highly functional and structural properties suitable for the final material applications.^17−21^

In the field of the rubber vulcanization process, employed for tire production, one main environmental concern is related to the use of ZnO, a fundamental curing activator that increases the sulfur vulcanization efficiency, by reducing reaction time, saving energy and costs of the whole process.^22−25^ ZnO is generally employed in conjunction with accelerators (i.e., sulfenamides or benzothiazoles) and coactivators (i.e., fatty acids as stearic acid, SA), in a complex sequence of multistep reactions, which lead to sulfur cross-link formation,^26−29^ through the following reaction mechanism: (i) ZnO reacts with SA to create highly reactive Zn(II)-SA adducts; (ii) then, Zn(II)-SA reacts with an accelerator and sulfur to form Zn complexes containing polysulfidic ligands, the active sulfurating agents; (iii) active sulfurating agents react with the polymer forming polysulfidic pendant cross-link intermediates; (iv) polysulfidic bridges form between the chains and progressively shorten through decomposition and rearrangement reactions promoted by Zn(II) centers, giving rise to highly cross-linked products. According to this mechanism, ZnO and especially Zn(II) centers have demonstrated to play a main role on both the kinetics of the curing reaction and the properties of vulcanized rubber NCs.^23,30−32^ However, due to the highly hydrophilic character of ZnO, opposed to rubber hydrophobicity, high oxide amounts are used in tire formulations in order to overcome its tendency to agglomerate and to guarantee a uniform cross-link formation inside the rubber matrix.

Moreover, several studies demonstrated that during the whole tire life cycle, Zn leaching occurs.^33,34^ This is mainly evident in urban areas due to tire tread consumption,^35,36^ leading to negative environmental effects. In fact, zinc concentrations even less than 1 mg/mL in the environment have been associated to toxicity and cytotoxicity, especially toward aquatic organisms.^37−40^ Considering that about 50% of the global ZnO annual production is used in the rubber industries for vulcanization in tire production,^41^ many authors claimed a large amount of zinc released by tires in the US and Europe.^42,43^ For instance, at the end of the nineties, the amount of zinc originated from tire tread and released to the environment was calculated to be about 150 tons per year only in both Sweden and Great Britain.^34,44^ According to these considerations, over the last few years, the reduction of the ZnO level in rubber formulations has become an urgent issue: the goal is to substitute or partially replace microcrystalline ZnO, conventionally used in the industrial curing process, without compromising the rubber materials’ performance.^24^

In the literature, different approaches have been proposed based on nanosized ZnO particles,^45−47^ zinc(II) complexes,^48,49^ or active zinc species supported on a matrix,^50−54^ aimed at increasing the Zn(II) center availability into the curing mechanism by increasing its dispersion in the matrix. Our group has proposed a novel activator, based on ZnO nanoparticles (NPs) directly grown on the surface of silica (ZnO/SiO_2_), that behaves at the same time as a vulcanization activator and a reinforcing filler.^55^ Thanks to the higher ZnO dispersion, ZnO/SiO_2_ enhanced the kinetics of the reaction and the efficiency of the curing process, through a reaction pathway that provides the formation of a highly reactive dinuclear Zn(II) complex coordinated by two bridged SA units,^32^ whose open structure enhances the availability of Zn centers to react with curative agents.

In this scenario, the present work aims at the development of a novel zinc-based curing activator, constituted by single-site Zn(II) centers, anchored onto the surface of SiO_2_ NPs. The objective is to obtain both fast kinetics and high curing efficiency in the vulcanization process, in order to reduce the total zinc content in rubber NC materials for a more sustainable production of tires.

The activator was designed based on three main concepts:

(i) realization of a double-function filler, i.e., an activator that simultaneously behaves as a reinforcing filler and a curing agent, where SiO_2_ NPs support the active sites providing their homogeneous distribution in the rubber matrix;
(ii) stability and fine dispersion of single metal atoms as active sites, achievable by anchoring the metal centers onto the SiO_2_ support through a covalent interaction with a ligand, thus preventing possible metal aggregation and leaching;
(iii) introduction of Zn(II) complexes with low steric hindrance, able to directly react with accelerators and sulfur to form an active sulfurating agent giving rise to polysulfidic cross-links according to the previously reported vulcanization mechanism.

In detail, SiO_2_ NPs were functionalized by an amino-substituted silane (3-aminopropyl-triethoxysilane, APTES), which provided the coordination through the amino groups of single Zn(II) centers onto the SiO_2_ surface. The structural, surface, and morphological analyses of amino silane-functionalized SiO_2_ NPs and of silica decorated with Zn(II) (ZnA-SiO_2_) were carried out to assess the most favorable structure and the amount of the single Zn(II) active sites, maximizing the Zn(II) availability for the interaction with the curative agents. To demonstrate the superior reactivity of the single-site active centers, ZnA-SiO_2_ samples were tested as an activator in the vulcanization reaction of isoprene rubber (IR), evaluating the curing efficiency and the dynamic mechanical properties of vulcanized NC materials, in comparison with those obtained by using conventional powdered ZnO. Finally, the model compound vulcanization (MCV) approach was employed to further investigate the curing mechanism with ZnA-SiO_2_.^56^ The results revealed the high stability of the Zn(II) single sites on SiO_2_ during the multistep vulcanization reaction along with the absence of metal leaching phenomena.

## 2 Experimental Section

### 2.1 Materials

For ZnA-SiO_2_ synthesis, precipitated SiO_2_ Rhodia Zeosil MP1165 (BET specific surface area, 160 m^2^ g^–1^) was obtained from Rhodia; (3-aminopropyl)triethoxysilane (H_2_N(CH_2_)_3_Si(OC_2_H_5_)_3_, APTES, 99%) was purchased from Sigma Aldrich; zinc nitrate hexahydrate (Zn(NO_3_)_2_·6H_2_O, 99%) and toluene (99%) were from Alfa Aesar; anhydrous ethanol (EtOH, 99.9%) was purchased from Exacta+Optech Labcenter.

For the preparation of rubber NCs, *cis*-1,4-polyisoprene rubber (IR) was purchased from Nizhnekamskneftekhim Export; bis(3-triethoxysilylpropyl)disulfide (TESPD) was from Aldrich; antioxidant *N*-(1,3-dimethylbutyl)-*N*′-phenyl-*p*-phenylenediamine (6PPD), Santoflex-6PPD, was from Flexsys. The curing agents were purchased as follows: SA (Stearina TP8) from Undesa; *N*-cyclohexyl-2-benzothiazole sulfenamide (CBS), Vulkacit CZ/X from Lanxess; sulfur Creso from Redball Superfine; ZnO (wurtzite, specific surface area, 5 m^2^ g^–1^) from Zincol Ossidi; *N*-cyclohexyl thiophthalimide (PVI) from Solutia.

For MCV analysis, 2,3-dimethyl-2-butene (TME, ≥98%), water for HPLC, and SA (98.5%) were obtained from Sigma Aldrich.

### 2.2 Synthesis and Characterization of the Zn Anchored SiO_2_ Activator

The curing activator ZnA-SiO_2_ was prepared following a two-step procedure. First, SiO_2_ NPs were functionalized by hydrolysis and condensation of APTES molecules. SiO_2_ (1.0 g) was dispersed under stirring in 24 mL of toluene for 10 min (120 °C). Then, suitable volumes of APTES (*V*_APTES_ equal to 0.202, 0.404, 0.606, and 1.212 mL), corresponding to APTES:SiO_2_ surface silanol molar ratios (*n*_APTES_/*n*_OH_) equal to 1:6, 1:3, 1:2, and 1:1, were added to the SiO_2_ suspension and kept under stirring for 24 h. The silanol amount of SiO_2_ was determined by thermogravimetric analysis (TGA) according to eq 1 in the Supporting Information and was equal to 5.2 mmol g^–1^. Finally, the suspension was cooled down at room temperature, and the powder was recovered by centrifugation, washed twice with fresh toluene, and dried at 80 °C overnight. Hereafter, APTES-functionalized samples are labeled A*_X_*-SiO_2_, where *X*, when reported, indicates the *n*_APTES_:*n*_OH_ ratio.

In the second step, Zn(II) centers were anchored to A-SiO_2_ through the coordination with the APTES amino groups. A-SiO_2_ (1.0 g) was dispersed under stirring in 50 mL of ethanol for 20 min at 100 °C to obtain a homogeneous suspension. Then, a suitable amount of Zn(NO_3_)_2_·6H_2_O was added, corresponding to Zn:amine molar ratios (*n*_Zn_/*n*_A_) equal to 1:2, 1:1, and 2:1. The amount of surface amine was determined by TGA according to eq 3 in the Supporting Information. The reaction was carried out for 2 h, and after cooling down, the powder was separated by centrifugation and washed twice with fresh ethanol, to eliminate the unreacted salt. Finally, the powder was dried at 80 °C for 12 h. Hereafter, these samples are labeled Zn*_Y_*A*_X_*-SiO_2_, where *Y*, when reported, indicates the *n*_Zn_/*n*_A_ ratio and *X* the *n*_APTES_:*n*_OH_ ratio.

As-prepared A*_X_*-SiO_2_ and Zn*_Y_*A*_X_*-SiO_2_ were fully characterized to confirm both the functionalization and the formation of the surface single-site zinc centers. Attenuated total reflectance Fourier transform infrared spectroscopy (ATR-FTIR) was performed using a PerkinElmer Spectrum 100 instrument (1 cm^–1^ resolution spectra, 650–4000 cm^–1^ region, 16 scans). Solid-state nuclear magnetic resonance (SS-NMR) was carried out by means of a Bruker 400WB spectrometer operating at a proton frequency of 400.13 MHz under the following conditions: (i) ^29^Si NMR: ^29^Si frequency of 79.48 MHz, cross polarization measurement (CP), contact time of 5 ms, π/2 pulse of 4 μs, and 2k scans; (ii) ^13^C NMR: ^13^C frequency of 100.52 MHz, CP, π/2 pulse of 3.5 μs, contact time of 2 ms, decoupling length of 6.3 μs, recycle delay of 4 s, and 2k scans; (iii) ^1^H NMR: ^1^H frequency of 400.13 MHz, π/2 pulse of 5 μs, recycle delay of 20 s, and 32 scans. Samples were packed in 4 mm zirconia rotors, which were spun at 7 kHz (10 kHz for ^1^H) under an air flow. Q_8_M_8_ and adamantane were used as external secondary references. Si units are labeled according to the usual NMR notation: T*^n^* and Q*^n^* represent trifunctional CSiO_3_ and tetrafunctional SiO_4_ units, respectively, and *n* is the number of oxo-bridges. The APTES amount in A*_X_*-SiO_2_ was evaluated by TGA using a TGA/DCS1 STARe system. The analyses were executed in the temperature range 30–1000 °C, at a constant air flow (50 mL min^–1^), and at a heating rate of 10 °C min^–1^; an isothermal step at 150 °C (15 min) was used to complete the weight loss due to physisorbed solvent molecules and water. The amount of linked APTES was estimated by the weight loss of A*_X_*-SiO_2_ in the range 150–1000 °C (Δ*W*_150–1000 °C_), mainly due to combustion of the functionalizing groups (−CH_2_CH_2_CH_2_NH_2_) corrected by the water desorption from residual surface silanol groups and from residual hydrolyzed APTES ethoxy groups not involved in bonding on SiO_2_ (calculated according to eqs 2–6 in the Supporting Information). The elemental CHNS analysis was performed to confirm the APTES quantification, using an Elementar VarioMICRO analyzer (temperature of the combustion column = 1150 °C, reduction column = 850 °C). The specific surface areas (SSA) of SiO_2_ NPs were measured before and after APTES functionalization by nitrogen physisorption using a Quantachrome Autosorb apparatus according to BET and BJH methods. Powder samples were evacuated at 200 °C for 16 h before the analysis.

Inductively coupled plasma–optical emission spectrometry (ICP-OES) was used to measure the amount of zinc in Zn*_Y_*A*_X_*-SiO_2_ using an ICP-OES Optima 7000 DV PerkinElmer instrument. Specimens for the analysis were prepared by finely grinding 0.20 g of powdered samples and dissolving them in a Teflon beaker with 4.0 mL of HNO_3_, 3.0 mL of HCl, and 1.0 mL of HF. The acid digestion was carried out in a microwave Milestone Ethos mineralizer instrument. Then, the sample was diluted with 12 mL of MQ water, centrifuged, and later further diluted to 1:100 to get optimal values for instrument detection. X-ray photoelectron spectroscopy (XPS) analysis was performed in a PerkinElmer Φ 5600-ci spectrometer using Al Kα radiation (1486.6 eV). The sample analysis area was 800 μm in diameter. Survey scans were obtained in the 0–1350 eV range (187.8 eV pass energy, 0.8 eV step^–1^, and 0.05 s step^–1^). Detailed scans were recorded for C 1s, O 1s, N 1s, Si 2p, and Zn 2p (23.5 eV pass energy, 0.1 eV step^–1^, and 0.1 s step^–1^). The standard deviation for the BE values is ±0.2 eV. The experimental uncertainty on the reported atomic composition values does not exceed ±5%. The XPS instrument was calibrated by assuming the binding energy (BE) of the Au 4f_7/2_ line at 83.9 eV with respect to the Fermi level. The BE shifts were corrected by assigning to the C 1s peak associated with adventitious hydrocarbons a value of 284.8 eV.^57^ Samples were mounted on steel holders and introduced directly in the fast-entry lock system of the XPS analytical chamber. The data analysis involved Shirley-type background subtraction, nonlinear least-squares curve fitting adopting Gaussian–Lorentzian peak shapes, and peak area determination by integration.^58^ The atomic compositions were evaluated from peak areas using sensitivity factors supplied by PerkinElmer, taking into account the geometric configuration of the apparatus.^59^

Lastly, an electron paramagnetic resonance (EPR) investigation was performed on Zn*_Y_*A*_X_*-SiO_2_ NPs, in which a small amount of Zn(II) centers was substituted by Cu(II) as a paramagnetic probe (Cu:Zn molar ratio = 1.0 × 10^–3^). The sample was prepared by substituting a suitable amount of Zn(NO_3_)_2_·6H_2_O with Cu(NO_3_)_2_·6H_2_O, following the same synthetic procedure. The aim was to have direct information on the number of APTES groups anchored to the SiO_2_ surface able to coordinate the single metal centers. The EPR spectra were acquired using a Bruker EMX spectrometer operating at the X-band frequency and equipped with an Oxford cryostat with the following conditions: 130 K, a modulation frequency of 100 kHz, a modulation amplitude of 5 G, and a microwave power of 5 mW.

### 2.3 Preparation of Silica/IR NCs

Silica/IR NCs were prepared by mixing IR, Zn*_Y_*A*_X_*-SiO_2_ as a filler and a curing activator, antioxidant 6-PPD, and the curing agents CBS and S_8_. In comparison with the common procedures to produce silica/rubber NCs, no addition of silane as a compatibilizing agent and powdered ZnO was carried out, as Zn*_Y_*-A*_X_*-SiO_2_ NPs are already functionalized with silane molecules coordinating zinc centers. Moreover, SA was not added as a coactivator since in Zn*_Y_*A*_X_*-SiO_2_ Zn(II) centers were supposed to be already available to react. NCs cured with Zn*_Y_*-A*_X_*-SiO_2_ were prepared with a silica constant content of 43.0 parts per hundred rubber (phr), while different zinc contents were tested by employing either Zn_1/2_-A_1/2_-SiO_2_ or Zn_1/2_-A_1/3_-SiO_2_ or Zn_1/2_-A_1/6_-SiO_2_, to get zinc contents equal to 1.5, 1.1, and 0.7 phr, respectively.

The ingredients were mixed in a Brabender Plasti-Corder lab station internal mixer (a 65 mL mixing chamber, a 0.6 filling factor, and 60 rpm rotor speed). The procedure of mixing can be divided into three steps: (1) IR was masticated into the mixing chamber at 90 °C, and Zn*_Y_*A*_X_*-SiO_2_ was added. After 3 min of mixing, required for the filler incorporation in the IR matrix, the antioxidant (6-PPD, 2.0 phr) was added. (2) The composites were reloaded into the mixing chamber at a temperature of 90 °C, and CBS (3.0 phr) and S_8_ (1.6 phr) were added (2 min of mixing). (3) The composites were further mixed in a two-roll mill at 50 °C for 3 min, to improve their homogeneity. Hereafter, the NCs will be called (*W*)ZnA-SiO_2_/IR where *W* indicates, when reported, the total Zn content in the composite (expressed in phr).

Reference SiO_2_/IR NCs were prepared using conventional microcrystalline ZnO as a curing activator. NCs were produced with a similar procedure to that previously described for (*W*)ZnA-SiO_2_/IR, by keeping constant the amount of both zinc and silica (43.0 phr). In the first step, bare SiO_2_ Zeosil 1165 (43.0 phr), TESPD (3.4 phr), 6-PPD (2.0 phr), ZnO, and the coactivator SA were mixed with IR at 145 °C; in the second step, the curing agents were added at a mixing temperature of 90 °C. Hereafter, the reference samples will be called (*W*)ZnO-SiO_2_/IR, where *W* indicates, when reported, the total Zn content in the composite (expressed in phr).

Lastly, technical IR NCs were prepared to verify the potential application of the ZnA-SiO_2_ activator in industrial tire compounds and its feasible use in the industrial production chain. Technical NCs (called ZnA-SiO_2_/IR-T and reference ZnO-SiO_2_/IR-T) were prepared with a Zn content of 1.5 phr and a SiO_2_ content of 43.0 phr (by using Zn_1/2_-A_1/2_-SiO_2_). The previous procedure was modified by implementing carbon black (CB, 15.0 phr) and wax (1.0 phr) in the first phase and a PVI retardant (0.4 phr) in the second phase. The ingredients were first mixed in an internal mixer (Pomini PL 1.6) for about 5 min (first step); as soon as the temperature reached 145 °C, the elastomeric blend was unloaded and further mixed with sulfur, CBS, and PVI in an open roll mixer (second step).

### 2.4 Vulcanization and Mechanical Properties of Silica/IR NCs

A rubber process analyzer (RPA2000, Alpha Technologies) was used to study the vulcanization curves and the dynamic mechanical properties of ZnA-SiO_2_/IR and ZnO-SiO_2_/IR. The composites were vulcanized at 170 °C and 100 bar for 5 min (frequency = 1.670 Hz, angle = 6.980°). The vulcanization curves were obtained by measuring viscosity over time with torque requested to keep the rotor at a constant rate. The dynamic mechanical properties were studied by applying a shear stress mode. The strain sweep tests were carried out at 70 °C and 10 Hz, at angle values between 0 and 10%. Specimens for this analysis were cut using a constant-volume rubber sample cutter (CUTTER 2000, Alpha Technologies); the dimensions were 3.5 cm diameter and 0.2 cm thickness; the weight was 5.0 ± 0.3 g. Two measurements were carried out for each sample, and the average value was reported. Swelling experiments were performed to measure the cross-linking densities of ZnA-SiO_2_/IR and ZnO-SiO_2_/IR. Samples of 20 × 20 × 3 mm^3^ (*m*_0_ = 1.00 ± 0.05 g) were immersed in closed vessels filled with 25 mL of toluene at 25 °C in the dark to avoid photodegradation reactions. The samples were swollen for four days, changing the solvent daily with fresh toluene, to eliminate the extracted fractions. At the end, the samples were dried for 24 h at room temperature, and the cross-linking densities were calculated according to eqs 7 and 8 as reported in the Supporting Information.

Technical NCs ZnA-SiO_2_/IR-T and ZnO-SiO_2_/IR-T were vulcanized with a rheometer Alpha Technologies type MDR2000. Two different vulcanization conditions were applied, either 30 min at 151 °C or 10 min at 170 °C, at an oscillation frequency of 1.66 Hz and an oscillation amplitude of ±0.5°. The morphological features of IR NCs were investigated by transmission electron microscopy (TEM) performed with a Zeiss EM 900 microscope operating at 80 kV. Ultrathin sections of pristine vulcanized sheets (frozen at −130 °C) were prepared with a Leica EM FCS cryo-ultramicrotome equipped with a diamond knife and collected on copper grids. Static and dynamic mechanical properties were measured after vulcanization at 151 °C: to study the elongation properties and handling of the tire, tensile tests were performed according to ISO38:2005. The tensile modulus at various elongation levels (10, 50, 100, and 300%, named CA0.1, CA0.5, CA1, and CA3) was measured using a ZwickRoell dumbbell tester, at a rate of 500 mm/min, at 23 °C. In addition, the dynamic mechanical properties were tested with an Instron dynamic device in compression and tension operation, at three different temperatures (10, 23, and 100 °C), measuring the dynamic elastic modulus (*E*′) and loss values (tan δ) when the samples were subjected to a dynamic sinusoidal tension (length = 25 mm, initial compression = 25% of longitudinal deformation with respect to its initial length, amplitude = ±3.5% with respect to the length of the preload, and frequency = 10 Hz).

### 2.5 Model Compound Vulcanization

The MCV approach was performed to study the structural changes of Zn*_Y_*A*_X_*-SiO_2_ during the curing reaction. MCV experiments were performed using TME as a model compound, cured at 120 °C as the optimal curing temperature,^32^ in the presence of Zn_1/2_A_1/2_-SiO_2_ (48.1 phr, equal to 43.0 phr of SiO_2_ and 1.5 phr of Zn) and the curing agents CBS (1.6 phr) and S_8_ (3.0 phr). No SA was added. MCV tests were repeated with microcrystalline ZnO, in the presence of bare SiO_2_, SA (2.0 phr), and curing agents, by keeping constant the silica and zinc contents (43.0 and 1.5 phr, respectively), under the same conditions. The curing reactions were performed in a 5 mL closed conical vial, immersed in an oil bath at 120 °C for different reaction times (5, 10, and 20 min). After the reaction, the vials were cooled down in a liquid nitrogen bath, and the mixture was filtered. The liquid phase (labeled as ZnA-SiO_2_/TME and ZnO-SiO_2_/TME depending on the used activator) was stored at 5 °C in the dark, to avoid evaporation and degradation phenomena of TME products, before being analyzed by ^1^H NMR to assess the completion of the reaction. ^1^H NMR spectra were recorded on a Bruker Avance 400 NEO spectrometer working at 500 MHz on samples dissolved in CDCl_3_ (1:10 vol:vol). Chemical shifts were determined relative to the residual solvent peak (CDCl_3_, δ 7.26 ppm). The solid phase (labeled as Zn_1/2_A_1/2_-SiO_2_/ER, where ER stands for the end of the reaction) was washed twice with fresh TME and ethanol, in order to remove traces of curing agents, and stored in air, before repeating XPS and SS-NMR analyses (see Paragraph 2.2). The same procedure was also repeated in the absence of sulfur, to study the structural changes due to the Zn(II)-CBS interaction (sample named Zn_1/2_A_1/2_-SiO_2_/CBS).

## 3 Results

### 3.1 Synthesis of the Zn-Anchored SiO_2_ activator—Zn*_Y_*A*_X_*-SiO_2_

#### 3.1.1 Characterization of A*_X_*-SiO_2_

The effective functionalization of SiO_2_ NPs with APTES was confirmed by ATR-FTIR and SS-NMR.

**Figure 1:**
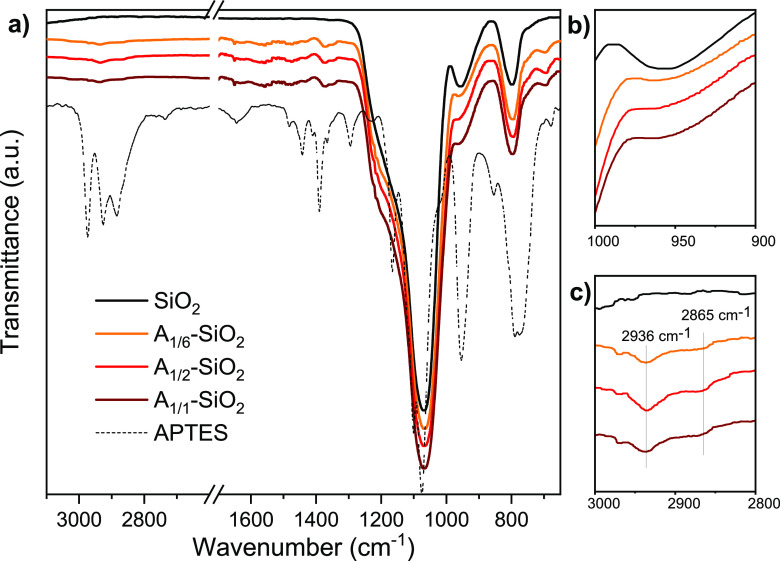
(a) FTIR spectra of A*_X_*-SiO_2_ compared with bare SiO_2_ and pure APTES. (b) Magnification of the ranges 900–1000 cm^–1^ and (c) 2800–3000 cm^–1^.

ATR-FTIR spectra of A*_X_*-SiO_2_ in comparison to bare SiO_2_ (Figure 1) show differences due to APTES molecules linked on the SiO_2_ surface: first, in A*_X_*-SiO_2_, the typical SiO_2_ peak at 1068 cm^–1^ due to the Si–O asymmetric stretching of the siloxane network and the peak at 958 cm^–1^ attributable to the Si–O stretching of surface silanols become larger,^55,60^ the latter decreasing in intensity and becoming a shoulder of the main Si–O peak due to the wider distribution of Si–O–Si angles and higher surface disorder. In addition, in A*_X_*-SiO_2_, the additional presence of the symmetric and asymmetric stretching of CH_2_ at 2865 and 2936 cm^–1^ is ascribable to the propyl chains of APTES. However, −NH_2_ bands are not evident in these spectra, probably due to their very low intensity compared to those of silica.

**Figure 2:**
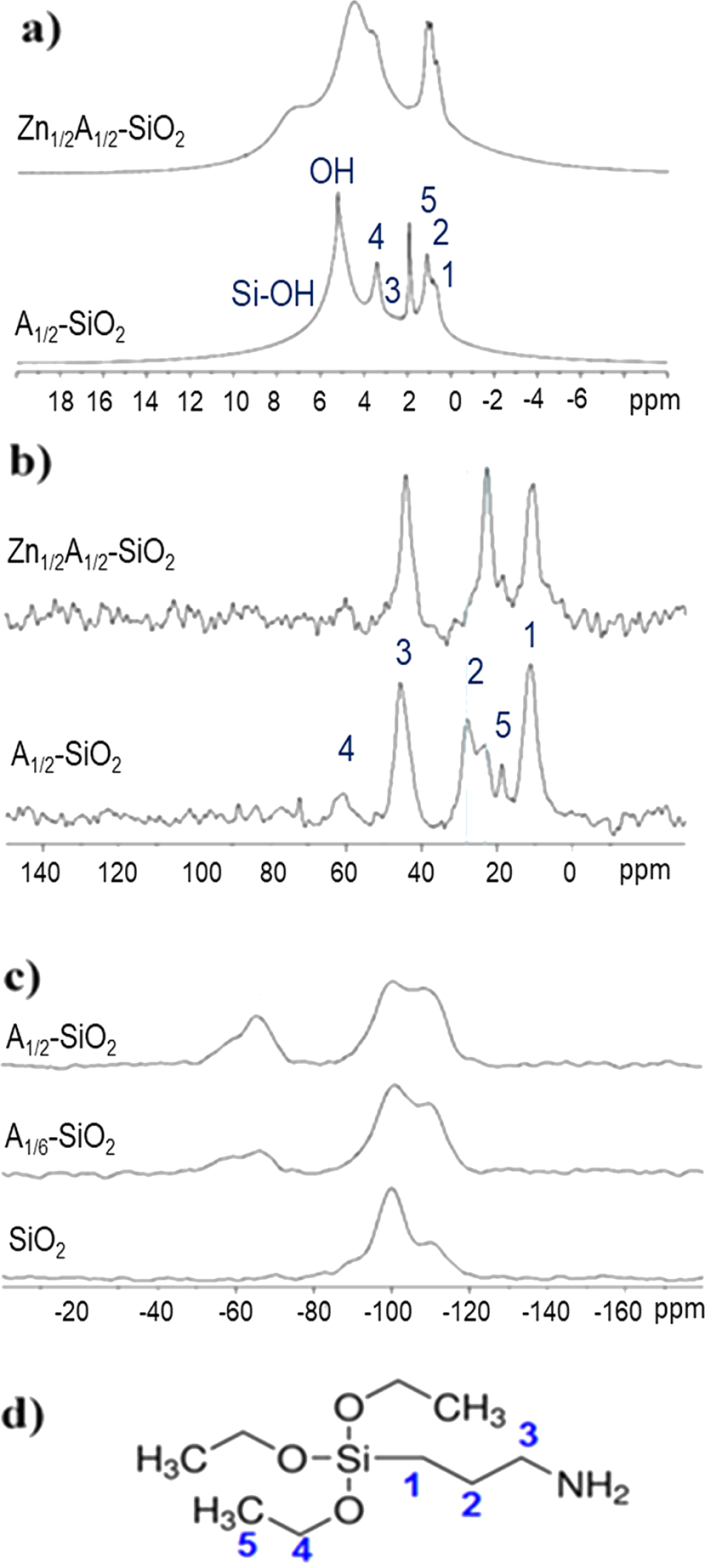
^1^H MAS (a) and ^13^C CPMAS (b) NMR spectra of Zn_1/2_A_1/2_-SiO_2_ and A_1/2_-SiO_2_; ^29^Si CPMAS (c) NMR spectra of A_1/6_-SiO_2_ and A_1/2_-SiO_2_ compared to the SiO_2_ one; carbon labeling of APTES (d).

The SS-NMR data on the same A*_X_*-SiO_2_ and bare SiO_2_ samples strengthen the above observations. ^29^Si CPMAS spectra of A*_X_*-SiO_2_ samples showed the typical signals due to Q^4^, Q^3^, and Q^2^ units of silica, respectively at −110, −100, and −92 ppm, and the T^3^ and T^2^ resonances at −66 and −57 ppm, related to the grafted APTES molecules. Figure 2c shows the spectra of A_1/2_-SiO_2_ and A_1/6_-SiO_2_ as examples.

Thanks to the good signal-to-noise ratio with respect to the related single-pulse experiments, the results of CP NMR can be used to define some trends, highlighted in Table 1 (the semiquantitative results of the profile fitting of ^29^Si CPMAS spectra are reported for the sake of completeness in Supporting Information, Table S1). In details: (i) the (Q^2^ + Q^3^)/Q^4^ intensity ratio decreases significantly from SiO_2_ to A*_X_*-SiO_2_ and by increasing *X*, evidencing the decrease of silica Si–OH groups due to surface functionalization; (ii) according to that, the total amount of T units increases with *X* as highlighted also by the T/Q ratio, which proportionally increases with the amount of grafted APTES; (iii) in addition, the T^2^/T^3^ ratio decreases with *X*, indicating an increase in the condensation degree. Finally, the small reduction of the Q^2^ and Q^3^ units, together with a broadening of the Q^2^ in A*_X_*-SiO_2_ with respect to bare SiO_2_, suggests that a partial Si–OH substitution with −OEt may occur.

**Table 1:** Relative Si Structural Unit Amounts of A*_X_*-SiO_2_ and SiO_2_ Samples Obtained from Line Shape Analysis of the ^29^Si CPMAS NMR Spectra (See Table S1)

sample			(Q^2^ + Q^3^)/Q^4^	T/Q	T^2^/T^3^
SiO_2_	100.0		2.7		
A_1/6_-SiO_2_	84.3	15.7	1.5	0.19	0.94
A_1/2_-SiO_2_	77.9	26.1	1.1	0.35	0.45

The above results were confirmed by ^13^C CPMAS spectra of A*_X_*-SiO_2_ (Figure 2b), thanks to the presence of the resonances due to APTES propyl chains (C1, C2, and C3 as labeled in Figure 2d), together with two low-intensity peaks due to the residual −OEt groups (C4 and C5 as labeled in Figure 2d). These signals give further structural information since the 10 ppm chemical shift of C1 indicates the successful condensation at the expense of the ethoxy groups, the C3 one downfield shifts with the amount of APTES (43.9 ppm for A_1/6_-SiO_2_ to 45.7 ppm for A_1/2_-SiO_2_), and finally, the C2 signal is formed by two components centered at about 28.1 and 23 ppm, with a rough 50:50 ratio. According to the literature,^61−63^ the C2 resonance is sensitive to the electronic environment of the terminal nitrogen that causes structural rearrangements and a propyl chain orientation as a consequence of the interaction with surface silanols. Thus, the downfield component can be due to less hydrated species, whereas the high-field 22 ppm resonance is due to hydrated ones obtained probably by proton transfer to the NH_2_ group from surface silanols.

Furthermore, the amount of aminopropyl groups on silica NPs was determined by TGA by the total weight loss percentage measured between 150 and 1000 °C (the thermal degradation profiles of bare SiO_2_ and the A*_X_*-SiO_2_ samples are reported in Figure S1 in the Supporting Information). The reaction yield of SiO_2_ functionalization with APTES (*Y*_A_), the total amount of APTES (*w*%_A_), and the number of APTES molecules over the SiO_2_ surface (molecules/nm^2^) are reported in Table 2 (according to eqs 4–6 in the Supporting Information).

**Table 2:** The Amount of APTES Anchored over SiO_2_ Calculated from TGA Results (According to Equations 2–6), Nitrogen Content (CHNS Analysis), and Specific Surface Areas (SSA) of A*_X_*-SiO_2_ Compared to Bare SiO_2_

	TGA					CHNS	
sample	Δ*W*_150–1000 °C_ [wt %]	*w*%_A_ [wt %]	*Y*_A_ [%]	no. of APTES molecules/SiO_2_ surface [molecules/nm^2^]	N* [wt %]	*N* [wt %]	SSA [m^2^ g^–1^]
SiO_2_	4.2 ± 0.1					0.03 ± 0.01	160
A_1/6_-SiO_2_	7.1 ± 0.1	3.2 ± 0.1	99	2.3 ± 0.1	0.75 ± 0.12	0.90 ± 0.15	107
A_1/3_-SiO_2_	8.0 ± 0.2	6.2 ± 0.2	95	4.4 ± 0.2	1.45 ± 0.28	1.38 ± 0.22	109
A_1/2_-SiO_2_	10.4 ± 0.2	7.6 ± 0.2	76	5.2 ± 0.2	1.78 ± 0.28	1.51 + 0.19	107
A_1/1_-SiO_2_	10.6 ± 0.3	8.4 ± 0.3	41	6.0 ± 0.3	1.97 ± 0.30	2.01 ± 0.31	106

The weight loss in A*_X_*-SiO_2_ increases with *X*, confirming the higher functionalization degree observed by NMR. However, the functionalization yield *Y*_A_ strongly decreases when the percentage of anchored APTES reaches the value of about 8 wt %. This depletion can be explained considering a saturation level of surface functionalization over that no more APTES molecules are able to bind to silica. Thus, ∼8% represents the maximum APTES coverage degree achievable onto the SiO_2_ surface, more likely due to steric hindrance and electrostatic repulsions between the functionalizing molecules, as already observed by Hicks et al.^64^ The number of APTES molecules anchored onto the silica surface, calculated considering the SiO_2_ SSA, ranges between two and six molecules on squared nanometers of the SiO_2_ surface depending on the amount of APTES used in the reaction (Table 2). These values indicate that the coverage of the SiO_2_ surface and the distance between the surface amino groups are suitable to act as coordination centers of zinc ions. CHNS analysis confirmed the quantification of the anchored APTES and the reaction yields (Table 2) since the measured nitrogen amount (N, wt %) was very similar to that contained in the APTES amount estimated by TGA (N*, wt %).

N_2_ physisorption analysis showed that the SSA of A*_X_*-SiO_2_ decreased compared to bare SiO_2_ (Table 2), in agreement with −OH consumption through functionalization with APTES molecules.

#### 3.1.2 Characterization of Zn*_Y_*A*_X_*-SiO_2_

Zn*_Y_*A*_X_*-SiO_2_ samples were characterized after the reaction of A*_X_*-SiO_2_ with the Zn(II) precursor. TGA and ATR-FTIR analyses (not shown) demonstrate that no silane loss occurred during the reaction. The T/Q and T^2^/T^3^ ratios calculated from the ^29^Si CPMAS spectrum confirm the structural stability of the inorganic core upon Zn grafting (Figure 2c). The amount of zinc anchored to SiO_2_ particles was determined by ICP-OES (Table 3) and depends on the functionalization degree of A*_X_*-SiO_2_. In fact, the molar ratios between anchored zinc and surface APTES molecules always resulted in 1:2, independently of the amount of the zinc precursor used in the synthesis (Table 3). Even if this observation is not direct information on the coordination sphere of the zinc centers, it strongly suggests that zinc centers are anchored to silica thanks to two silane molecules through the coordination with the APTES amine groups.

**Table 3:** The Amount of Zinc Anchored to the SiO_2_ Surface Measured by ICP-OES

	synthesis	ICP-OES		
sample	(*y*) nominal *n*_Zn_:*n*_APTES_ [mol:mol]	Zn amount [wt %]	no. of Zn atoms/surface [atoms/nm^2^]	*n*_Zn_:*n*_APTES_ [mol:mol]
Zn*_Y_*A_1/6_-SiO_2_	1:2	1.6 ± 0.1	1.2 ± 0.1	0.52
	1:1	1.7 ± 0.1	1.3 ± 0.1	0.56
	2:1	1.6 ± 0.1	1.2 ± 0.1	0.52
Zn*_Y_*A_1/3_-SiO_2_	1:2	2.0 ± 0.2	1.8 ± 0.2	0.41
	1:1	2.2 ± 0.1	2.0 ± 0.1	0.46
	2:1	2.3 ± 0.1	2.1 ± 0.1	0.48
Zn*_Y_*A_1/2_-SiO_2_	1:2	2.9 ± 0.2	2.4 ± 0.2	0.46
	1:1	3.2 ± 0.2	2.6 ± 0.2	0.50
	2:1	3.3 ± 0.1	2.7 ± 0.1	0.52

The effective coordination of zinc ions by APTES amine groups was supported by both the ^13^C CPMAS and ^1^H MAS NMR analyses. The carbon spectrum of Zn_1/2_A_1/2_-SiO_2_ samples, chosen as a representative example, shows the presence of only one component of the C2 signal at 22 ppm, instead of the two signals discussed for the A_1/2_-SiO_2_ sample (Figure 2b). This strongly suggests that the terminal amino groups are mainly coordinated to zinc centers rather than being involved in the interaction with surface silanols (“hydrated component”). Moreover, the ^1^H MAS spectra of Zn_1/2_A_1/2_-SiO_2_ and A_1/2_-SiO_2_ are different even if a precise peak assignment is quite difficult due to the broadening induced by the typical strong ^1^H–^1^H homonuclear dipolar coupling (Figure 2a).

Anyway, according to the literature,^65^ in the A*_X_*-SiO_2_ spectra, it is possible to attribute (i) the overlapped peaks at 0.7, 1.0, and 2.6 ppm to methylene protons of the propyl chains **1**, **2**, and **3**, respectively, and the two resonances at 1.1 and 3.4 ppm to ethoxy groups, both nonhydrolyzed and due to Si–OH substitution (as discussed in 3.1.1); (ii) the sharp peak at 5.2 ppm to free −OH, whereas the broad band at 4 ppm to −OH due to adsorbed water; (iii) the sharp resonance at 1.9 ppm to surface-isolated Si–OH;^66^ (iv) the broad shoulder at 7.3 ppm to interacting Si–OH, such as in bare SiO_2_ (Figure S2 in the Supporting Information), and the broad component at about 0.4 ppm to −OH with a different acidity. However, both the latter two resonances can be related also to amino species such as −NH_3_^+^ and −NH_2_, respectively, in agreement with the line shape of C2 resonance in the ^13^C CPMAS spectrum, previously discussed.^67,68^ The same pattern was detected for Zn*_Y_*A_1/2_-SiO_2_ proton spectra, except for the disappearance of the 1.9 and 5.2 ppm sharp peaks and the increase of both the bands at 7.3 and 4.6 ppm. In agreement with Kim et al.,^69^ this behavior can be related to the interaction of the zinc ions with the amino groups, thus confirming their effective coordination with the anchored APTES molecules.

The surface atomic compositions of Zn*_Y_*A*_X_*-SiO_2_, A*_X_*-SiO_2_, and SiO_2_ were assessed by XPS analysis (Table 4). Compared to bare SiO_2_, in A_1/2_-SiO_2_ and Zn_1/2_A_1/2_-SiO_2_, the photoemission peak of N 1s was observed. Moreover, in agreement with the silica functionalization with APTES, a higher amount of carbon was detected. In addition, in Zn_1/2_A _1/2_-SiO_2_, the Zn 2p peak confirms the zinc coordination onto the functionalized silica surface.

**Table 4:** Surface Atomic Percentage of Zn*_Y_*A*_X_*-SiO_2_, A*_X_*-SiO_2_, and SiO_2_ Measured by XPS

samples	C %	O %	Si %	Zn %	N %	NH %	NO_3_ %	Zn:NH	Zn:NO_3_
SiO_2_	3.2	70.9	26.0						
A_1/2_-SiO_2_	19.6	53.4	22.2		4.8	4.8			
Zn_1/2_A_1/2_-SiO_2_	16.1	55.2	20.1	2.6	6.1	4.3	1.7	0.6	1.5

**Figure 3:**
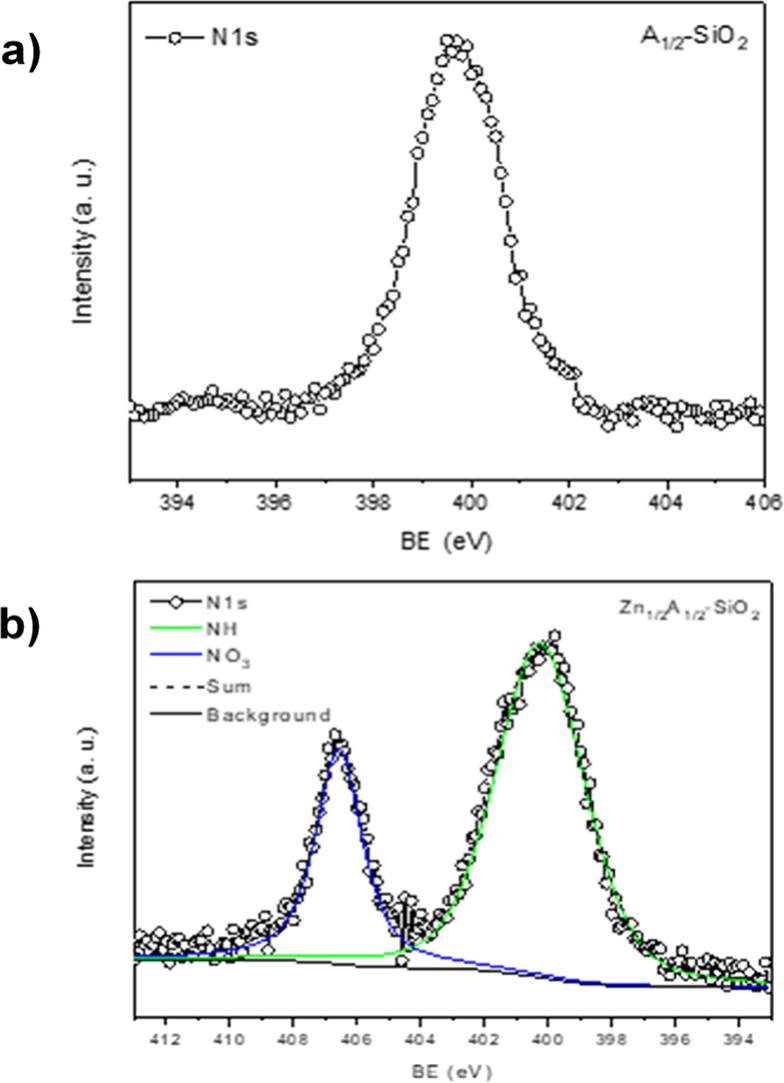
N 1s photoemission peaks of (a) A_1/2_-SiO_2_ and (b) Zn_1/2_A_1/2_-SiO_2_.

The N 1s peak deserves further comments. In A_1/2_-SiO_2_ (Figure 3a), the signal shows only one component (BE = 399.7, labeled NH) ascribed to N atoms in an organic environment compatible with the APTES amino groups. On the other hand, in Zn_1/2_A_1/2_-SiO_2_ (Figure 3b), the N 1s peak clearly shows two main components, the contribution given by the APTES amino groups (NH, BE = 400.0 eV) and an inorganic one due to nitrate groups (BE = 406.7 eV, labeled as NO_3_). This latter contribution, originated from the zinc precursor, is more likely due to the coordination of nitrate groups to the zinc centers anchored on the silica surface.

**Figure 4:**
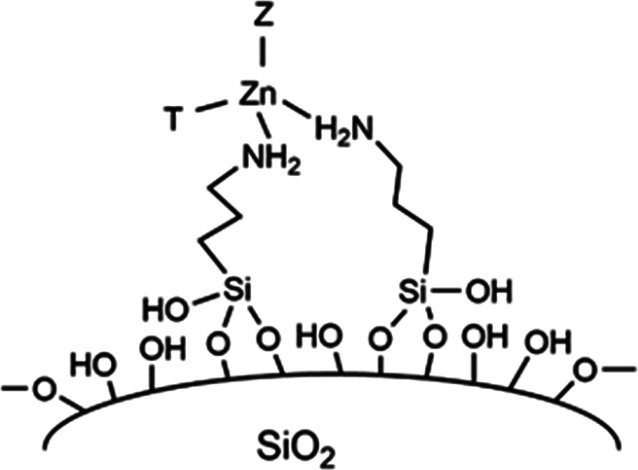
Sketch of the single-site Zn(II) centers anchored to silica in Zn*_Y_*A*_X_*-SiO_2_ samples. T and Z may correspond to groups coordinated to Zn(II), as NO_3_^–^ or OH^–^.

The value of the Zn/NH ratio derived from XPS (Table 4) is similar to that obtained from ICP measurement for the same sample, thus validating the structure of the surface Zn(II) centers bonded to two amine groups of APTES (Figure 4).

In order to have both direct evidence of the ability of the APTES groups anchored to the SiO_2_ surface to coordinate single metal centers and an estimation of the number of functional groups, which coordinate every single metal center, the (Cu)Zn_1/2_A_1/2_-SiO_2_ sample was prepared by substituting a small amount of Zn(II) centers with the paramagnetic probes Cu(II) (Cu:Zn 1:1000) and investigating it by EPR spectroscopy. EPR spectra of (Cu)Zn_1/2_A_1/2_-SiO_2_ show the resonance line attributed to a monomeric Cu(II) species in axial symmetry with *g*_∥_ = 2.26 and *g*_⊥_ = 2.06 with a hyperfine coupling constant *A*_∥_ = 185 G (Figure S3 in the Supporting Information). The magnetic tensor values are consistent with tetragonally elongated or square-planar or square pyramidal ions with two nitrogen and two oxygen ligands in the equatorial positions.^70,71^ These results indicate that Cu(II) centers are anchored to silica as isolated copper centers, without any metal–metal interactions; in addition, only two nitrogen ligands are supposed to bond every single Cu(II) site, confirming that APTES molecules are close enough to coordinate one single metal center and that no more than two APTES ligands are available for each center, neither from the same SiO_2_ particle nor from other vicinal particles. The presence of two equatorial oxygen ligands suggests the possible interaction of these centers with other species, such as water molecules, hydroxyl groups, or nitrate groups (through the oxygen atom), as previously shown by XPS.

In conclusion, the characterization of ZnA-SiO_2_ has shown that the material is likely composed of isolated Zn(II) single sites anchored to the SiO_2_ surface through the coordination of zinc centers with two amino groups, as represented in the Figure 4, and that the coordination sphere of Zn ions, more likely in the most common tetrahedral symmetry,^72^ is partially occupied by NO_3_^–^ ions or possibly water/hydroxy groups. These are labile terminations, which can be easily exchanged, suggesting that such coordination sites could possibly be occupied by other species originated in the catalytic process. As the Zn loading can be suitably controlled by the SiO_2_ functionalization degree with APTES, Zn*_Y_*A*_X_*-SiO_2_ samples were tested as activators for IR vulcanization, allowing preparation of IR NCs at different Zn contents.

### 3.2 Silica/IR NCs

(*W*)ZnA-SiO_2_/IR samples were compounded and vulcanized at 170 °C using Zn*_Y_*A*_X_*-SiO_2_ both as a curing activator and a reinforcing filler. (1.5)ZnA-SiO_2_/IR was compared with the (1.5)ZnO-SiO_2_/IR reference material, conventionally prepared using microcrystalline ZnO and SA, as a curing activator and a coactivator, in addition to the compatibilizing TESPD agent.

**Figure 5:**
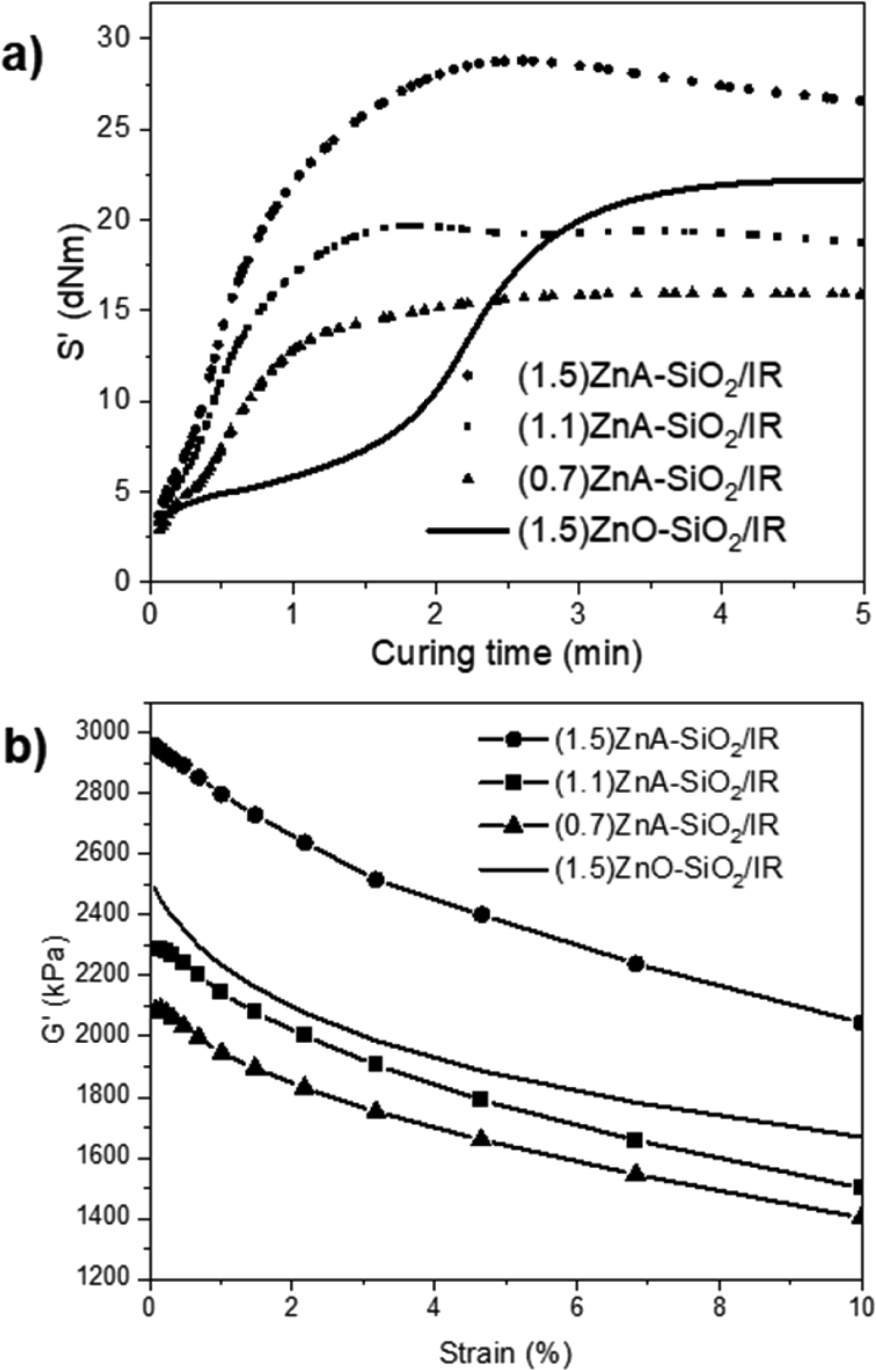
Comparison of the vulcanization curves (a) and of the elastic modulus *G*′ (b, 0–10% strain) measured for SiO_2_/IR NCs cured with Zn*_Y_*A*_X_*-SiO_2_ at different Zn contents: (1.5)ZnA-SiO_2_/IR (circles), (1.1)ZnA-SiO_2_/IR (squares), and (0.7)ZnA-SiO_2_/IR (triangles). Vulcanization and *G*′ curves of (1.5)ZnO-SiO_2_/IR NCs (solid line) are also reported in (a) and (b), respectively, as a reference sample.

The vulcanization curves of both ZnA-SiO_2_/IR and ZnO-SiO_2_/IR were registered by measuring the torque (*S*′) values over the curing time. The *S*′ increase was due to sulfur cross-link formation between polymer chains, responsible for the higher viscosity of the vulcanized material. Comparing the curves of (1.5)ZnA-SiO_2_/IR and (1.5)ZnO-SiO_2_/IR at the same zinc content, the improvements of the vulcanization rate and efficiency due to the use of Zn*_Y_*A*_X_*-SiO_2_ substituting microcrystalline ZnO are demonstrated by (Figure 5a and Table 5) (i) the higher maximum torque (*M*_ma*x*_, the torque measured when the sulfur reticulation is complete), (ii) the lower optimum curing time (*t*_90_, the time required for reaching 90% of *M*_ma*x*_ at the curing temperature), and (iii) the lower scorch time (*t*_S1_, the time to reach the first sign of incipient cross-linking). The increased performance of Zn*_Y_*A*_X_*-SiO_2_ was evident also at the lowest tested Zn content, ((1.1)ZnA-SiO_2_/IR and (0.7)ZnA-SiO_2_/IR, Figure 5a), even if the significant reduction of the activator caused a lower vulcanization efficiency. A partial reversion of vulcanization (overcuring) occurs for Zn*_Y_*A*_X_*-SiO_2_-IR NCs continuing the thermal treatment after *M*_max_, as typically expected due to the partial desulfurization process of vulcanized IR NCs after the optimum curing time (*t*_S1_ particularly low for ZnA-SiO_2_-IR, Table 5).

**Table 5:** Vulcanization Parameters and Mechanical Properties of (*W*)ZnA-SiO_2_/IR and (*W*)ZnO-SiO_2_/IR NCs: *M*_min_ = Minimum Torque; *M*_max_ = Maximum Torque; *t*_S1_ = Scorch Time; *t*_90_ = Time to Achieve 90% of *M*_max_; *G*′_0_ = Elastic Modulus at Low Strain (0.1%); *G*′_10%_ = Elastic Modulus at 10% Strain; Δ*G*′ = *G*′_0_ – *G*′_10%_

sample	*M*_min_ [dNm]	*t*_S1_ [min]	*M*_max_ [dNm]	*t*_90_ [min]	*G*′_0_ [kPa]	*G*′_10%_ [kPa]	Δ*G*′ [kPa]
(1.5)ZnA-SiO_2_/IR	3.7	0.1	28.8	1.6	2958	2044	914
(1.5)ZnO-SiO_2_/IR	3.3	0.6	22.2	3.1	2489	1668	821
(1.1)ZnA-SiO_2_/IR	3.2	0.1	19.6	1.2	2283	1501	782
(0.7)ZnA-SiO_2_/IR	2.9	0.1	16.0	1.7	2082	1402	680

These data support the high Zn availability of the Zn centers in Zn*_Y_*A*_X_*-SiO_2_ to react with the curing agents in the first vulcanization stages without adding the coactivator (SA). In fact, in an accelerated sulfur vulcanization process, the *t*_S1_ value is associated with the time required by the zinc activator and curing agents to form intermediate reactive species, necessary to favor the subsequent sulfur–polymer interaction and cross-link formation.^22^ In ZnO-SiO_2_/IR NCs, the first reaction step is the complexation and solubilization of Zn(II) centers given by the interaction between the ZnO activator and the SA coactivator followed by the formation of an active sulfurating agent; whereas, in Zn*_Y_*A*_X_*-SiO_2_, the single-site Zn centers anchored on the SiO_2_ surface are already distributed in the matrix and prone to the reaction with the curatives, bypassing the first Zn(II) complexation step, so that the total curing reaction is faster. The direct impact of single-site zinc centers on the kinetics of the process does not result only in a faster reaction rate and in a shorter initial delay but also in a more efficient cross-linking formation demonstrated by the high *M*_max_.

The vulcanized NCs ZnA-SiO_2_ and ZnO-SiO_2_/IR were morphologically investigated by TEM performed on thin slices of the IR NCs (Figure S4 in the Supporting Information). The analysis revealed that SiO_2_ particle sizes are similar in both samples confirming that the surface treatments on SiO_2_ with both APTES and the Zn precursor did not affect the SiO_2_ particle dimensions. In addition, ZnA-SiO_2_ was shown to be well-dispersed in IR, promoting the formation of the SiO_2_ network, without any filler segregation and comparable to ZnO-SiO_2_/IR, suggesting that also in the absence of TESPT as a compatibilizing agent, APTES linked on the SiO_2_ surface makes nanoparticles suitable to be dispersed in rubber.

The higher curing efficiency of Zn*_Y_*A*_X_*-SiO_2_ was confirmed also by the cross-linking densities of (1.5)ZnA-SiO_2_/IR and (1.5)ZnO-SiO_2_/IR NCs, evaluated through swelling experiments, according to the Flory–Rehner equation (eqs 7 and 8 in the Supporting Information).^73^ In agreement with the vulcanization curves, the cross-linking density of (1.5)ZnA-SiO_2_/IR (6.3 × 10^–5^ mol g^–1^) is higher than that of (1.5)ZnO-SiO_2_/IR NCs (3.2 × 10^–5^ mol g^–1^).

These results stated that both Zn*_Y_*A*_X_*-SiO_2_ and conventional ZnO materials play the role of an activator in the vulcanization process and that the highly reactive zinc single sites in Zn*_Y_*A*_X_*-SiO_2_ promote a more efficient cross-link formation compared to ZnO, confirming that Zn*_Y_*A*_X_*-SiO_2_ may be used to either increase the performances of the process or to reduce the total Zn amount in rubber NCs. In addition, the different locations of the zinc active sites in Zn*_Y_*A*_X_*-SiO_2_ close to the filler particles suggest that the morphological distribution of the sulfur cross-links in the rubber may be different from ZnO-SiO_2_/IR, where sulfurating complexes form in the rubber,^74,75^ even if not detrimental regarding the performances.

The vulcanization process strongly modifies the dynamic mechanical behavior and the reinforcement of rubber NCs. Thus, a more efficient vulcanization due to the different curing activation should further enhance the properties of the final composite material. The dynamic mechanical analyses of cured (1.5)ZnA-SiO_2_/IR and (1.5)ZnO-SiO_2_/IR NCs were carried out measuring the change in the elastic modulus (*G*′) *vs* the strain in the range 0–10% (Figure 5b), in the presence of the same amount of the reinforcing silica filler (43.0 phr). In agreement with the mechanical improvement highlighted by vulcanization curves, the highest reinforcement and filler–rubber interactions were measured for (1.5)ZnA-SiO_2_/IR compared to (1.5)ZnO-SiO_2_/IR, as demonstrated by the highest values of *G*′_0_ and *G*′_10%_ the *G*′ modulus measured at low 0.1% and high 10% strain, respectively (Table 5). Particularly, the increases in both *G*′_0_ and *G*′_10%_ and the slight increase in Δ*G*′ evidence that the enhancement of *G*′ is only partially due to the strain-dependent contribution to the modulus due to the filler network and mainly due to the strain-independent contribution, related to the more efficient cross-linked polymer network.^76^

Considering that the hydrodynamic effect is similar for both samples due to the equal amount of the filler, it cannot be excluded that also a different filler–rubber interaction may affect the strain-independent contribution due to the different surface modification of the filler caused by the compatibilizing agent TESPT in ZnO-SiO_2_/IR and the anchored APTES in ZnA-SiO_2_/IR. However, the results demonstrate that such different interaction with the rubber in ZnA-SiO_2_ has no detrimental effects on the curing process and the mechanical properties of the composite material. The reduced *G*′ values of (1.1)ZnA-SiO_2_/IR and (0.7)ZnA-SiO_2_/IR were consistent with the lower Zn contents compared to (1.5)ZnA-SiO_2_/IR, even though with similar trends to (1.5)ZnO-SiO_2_/IR. These data further suggested that Zn*_Y_*A*_X_*-SiO_2_ can promote an efficient cross-link formation by using lower Zn amounts, compared to microcrystalline ZnO, with a potential beneficial effect in terms of reduction of Zn usage.

The activity of ZnA-SiO_2_ as a curing activator was also tested in the vulcanization of technical IR NCs.

The vulcanization curves and the mechanical properties of ZnA-SiO_2_/IR-T further highlighted the greater reactivity of ZnA-SiO_2_/IR compared to microcrystalline ZnO. In fact, the higher *M*_max_ values of ZnA-SiO_2_/IR-T (about 50% higher) along with a 30% reduction of *t*_90_ than obtained for ZnO-SiO_2_/IR-T (Table S2 of the Supporting Information) confirmed a higher process efficiency.

In addition, ZnA-SiO_2_/IR-T shows a higher elongation modulus at 10 and 50% (CA0.1 and CA0.5 in Table S3 of the Supporting Information) up to 100% (CA1.0) values very similar for both samples, resulting in higher stiffness at lower strains and lower elongation at break (CR), probably related to the highest sulfur cross-link density.

Also, the *E*′ values of ZnA-SiO_2_/IR-T from dynamic mechanical measurement at different temperatures (Table S3 of the Supporting Information) are always higher than those of ZnO-SiO_2_/IR-T, confirming that ZnA-SiO_2_ could be used as a valid substituent to reduce the Zn amount without affecting the mechanical performances of the vulcanized compound. Actually, tan δ values are slightly less favorable in ZnA-SiO_2_/IR-T, but this could be related to the absence of any compatibilizing agent as TESPT, which strongly improves the filler–rubber interaction of silica.

### 3.3 MCV

**Scheme 1:**
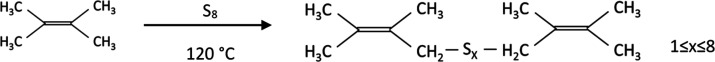
Reaction of TME with S_8_ Producing Cross-Linking Products with Different Lengths of Sulfur Bridges (6 ≤ *X* ≤ 8)

The MCV approach was used to investigate the reaction mechanism and the structural stability of Zn(II) centers in Zn*_Y_*A*_X_*-SiO_2_ along the reaction pathway, by performing the vulcanization of the model TME monomer. TME behaves as an unsaturated polymer toward sulfur, able to mimic IR reactivity.^77,78^ Sulfur bridges are supposed to form in-between TME molecules, through the allylic positions of the methyl groups (Scheme 1).

**Figure 6:**
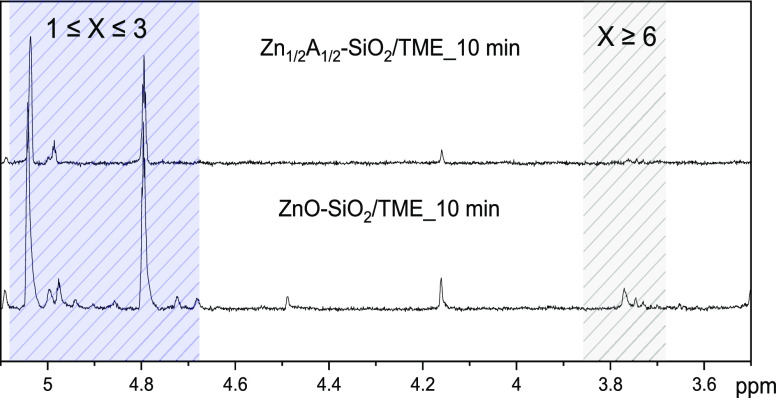
^1^H NMR spectra (range, 3.5–5.1 ppm) of Zn_1/2_A_1/2_-SiO_2_/TME and ZnO-SiO_2_/TME after 10 min of reaction time.

First, the vulcanized liquid Zn*_Y_*A*_X_*-SiO_2_/TME product was analyzed through ^1^H NMR to confirm the formation of the cross-link products at different reaction times up to the optimum curing time (5, 10, and 20 min) and compared to ZnO-SiO_2_/TME. Figure 6 shows the ^1^H NMR spectra (range, 3.5–5.1 ppm) of Zn_1/2_A_1/2_-SiO_2_/TME and ZnO-SiO_2_/TME after 10 min of reaction (^1^H NMR spectra at 5 and 20 min are reported in Figure S5 in the Supporting Information). In all ^1^H NMR spectra of Zn_1/2_A_1/2_-SiO_2_/TME and ZnO-SiO_2_/TME, the signals at 4.73 and 4.83 ppm were associated to the formation of isomeric cross-linked TME mono- or disulfide products, which form via allylic substitution during the curing process.^79^ In addition, the signals at 5.07 and 4.97 ppm were ascribed to a partial oxidation of TME and to the subsequent formation of 2,3-dimetylbutadiene.^56^ Additional peaks in the range of 3.4–3.7 ppm were detected for both Zn_1/2_A_1/2_-SiO_2_/TME and ZnO-SiO_2_/TME at the beginning of the reaction (5 min), assigned to TME-S*_X_*-TME products with *X* ≥ 6. However, these peaks completely disappeared in Zn_1/2_A_1/2_-SiO_2_/TME after 10 min (Figure 6), whereas they persisted in ZnO-SiO_2_/TME at 10 min. These observations confirmed that TME undergoes the vulcanization reaction under the applied experimental conditions, through a progressive shortening of the sulfur bridges until the formation of the shortest ones (1 ≤ *X* ≤ 3), as already observed in previous studies.^22,32^ Moreover, the ^1^H NMR data suggest that Zn_1/2_A_1/2_-SiO_2_ strongly affects the reaction kinetics, leading to the formation of shorter sulfur bridges at lower reaction times, in agreement with the vulcanization curves of IR NCs and supporting the higher availability and reactivity of the single-site Zn(II) centers.

To understand the structural changes of Zn(II) centers, Zn*_Y_*A*_X_*-SiO_2_ powders were collected and analyzed by ^1^H MAS NMR and XPS after the consecutive addition steps of curing agents: (a) accelerator CBS added to Zn*_Y_*A*_X_*-SiO_2_/TME solution at 120 °C, 20 min (Zn*_Y_*A*_X_*-SiO_2_/CBS); (b) S_8_ added to the previous solution and vulcanization at the optimum curing time, 20 min (Zn_1/2_A_1/2_-SiO_2_/ER). Their surface properties were compared to the previously described features of Zn_1/2_A_1/2_-SiO_2_.

^1^H MAS NMR spectra of Zn_1/2_A_1/2_-SiO_2_/ER, compared with those of Zn_1/2_A_1/2_-SiO_2_ before the reaction, show similar patterns (Figure S6 in the Supporting Information), suggesting that the structure of Zn(II) centers does not change significantly after interacting with CBS and S_8_. In fact, the broad peak at 7.3 ppm due to the protons of −NH_2_ groups coordinated to zinc ions, already observed in Zn*_Y_*A*_X_*-SiO_2_ after the metal coordination, was maintained. Moreover, the relative intensities of the peaks due to the functionalizing agent were preserved, supporting the stability of the functionalizing agents over SiO_2_ during the process.

XPS analysis was performed on Zn_1/2_A_1/2_-SiO_2_/ER and Zn_1/2_A_1/2_-SiO_2_/CBS and compared with Zn_1/2_A_1/2_-SiO_2_. The surface atomic percentages are reported in Table 6.

**Table 6:** Surface Atomic Percentage of Zn_1/2_A_1/2_-SiO_2_, Zn_1/2_A_1/2_-SiO_2_/CBS, and Zn_1/2_A_1/2_-SiO_2_/ER Measured by XPS

samples	C %	O %	Si %	Zn %	N tot. %	NH %	NO_3_ %	Zn:NH	Zn:NO_3_
Zn_1/2_A_1/2_-SiO_2_	16.1	55.2	20.1	2.6	6.1	4.3	1.7	0.6	1.5
Zn_1/2_A_1/2_-SiO_2_/CBS	18.5	53.8	19.4	2.8	5.6	4.3	1.3	0.6	2.2
Zn_1/2_A_1/2_-SiO_2_/ER	20.0	52.6	19.6	2.7	5.1	3.9	1.2	0.7	2.3

The results evidence that Zn and amino nitrogen amounts (Zn and NH, Table 6) remain quite constant on all samples, confirming that Zn(II) centers are stable during the curing reaction and no Zn release occurs after the interaction with the curing agents. On the contrary, the amount of nitrate nitrogen (NO_3_, Table 6) decreases both in Zn_1/2_A_1/2_-SiO_2_/CBS and in Zn_1/2_A_1/2_-SiO_2_/ER, indicating that nitrate groups on Zn(II) centers may be labile enough to be partially exchanged by CBS molecules during the first step of the curing reaction, giving rise to the formation of intermediate Zn(II)-based complexes. The quantification of the sulfur amount on both Zn_1/2_A_1/2_-SiO_2_/CBS and Zn_1/2_A_1/2_-SiO_2_/ER surfaces resulted in the presence of traces (<0.2%), revealing that the interaction of Zn(II) with CBS and S_8_ probably gives rise to sulfurating intermediate species highly reactive toward TME molecules and not stable enough to be detected on the surface of the activator.

Both ^1^H MAS NMR and XPS results suggest that Zn(II) centers behave as heterogeneous catalytic sites on the SiO_2_ surface, as indicated by the constant Zn:NH molar ratios along the pathway of the curing reaction, through a mechanism that implies exchange reactions with labile groups as NO_3_ bonded to Zn(II) centers. The stability of the Zn(II) centers anchored to SiO_2_ is particularly relevant since it is connected to a lower zinc leaching, which is a common problem of the processing and using of vulcanized rubber composites, especially for tire applications.

## 4 Conclusions

In this work, the design and preparation of an innovative Zn(II)-based activator for rubber vulcanization ZnA-SiO_2_, constituted by Zn(II) single sites anchored on the surface of SiO_2_ nanoparticles (NPs), were reported.

ZnA-SiO_2_ was synthesized through the SiO_2_ surface functionalization with amino silane followed by the coordination of Zn(II) ions through the amino groups. The control of the Zn loading was achieved by suitably tuning the functionalization degree of silica, keeping constant the zinc:amino molar ratio and equal to 1:2. The coordination sphere of anchored Zn centers is completed by NO_3_^–^ or other labile groups, e.g., OH^–^ and water ions, able to be exchanged with the curing agents during the vulcanization reaction.

ZnA-SiO_2_ was applied as an activator for the vulcanization of IR NCs, behaving as a double-function filler, at the same time acting as a rubber reinforcing agent and a curing activator. It demonstrated higher reactivity of Zn(II) centers and efficiency in the curing process compared to microcrystalline ZnO, conventionally employed as an activator for industrial rubber vulcanization, with a strong impact both on the reaction kinetics and on the dynamic mechanical properties of the vulcanized NC material. In particular, the higher cross-linking densities and the enhanced elastic modulus confirmed the highly efficient assembly of the sulfur-polymer network inside the rubber matrix.

ZnA-SiO_2_ represents a promising material to improve the sustainability of the industrial vulcanization process and production of rubber NCs. By introducing more disperse and active zinc species in the process, the amount of employed zinc can be reduced compared to conventional microcrystalline ZnO, keeping high both curing efficiency and mechanical behavior of the final NC material. In addition, the high structural stability of Zn(II) centers, linked to silica during the curing reaction, may hinder Zn leaching from rubber products, with less environmental impact on the aquatic ecosystem.

The results highlight that the proposed activator may be a good candidate to substitute the conventional ZnO activator in the vulcanization process to produce less environmental impact of rubber-based materials.

## Supporting Information Available

Thermal degradation profiles of A*_X_*-SiO_2_, mathematical elaboration of TGA data (eqs 1–6), profile fitting results in the ^29^Si CPMAS NMR spectra, ^1^H MAS NMR spectrum of SiO_2_, EPR spectrum of Zn_1/2_A_1/2_-SiO_2_, mathematical elaboration of swelling experiments (Flory–Rehner approach, eqs 7 and 8), ^1^H NMR recorded during the MCV approach at two reaction times, ^1^H MAS NMR spectral comparison of Zn_1/2_A_1/2_-SiO_2_ before and after the MCV reaction (PDF)
