# Supplementary material for: Design of a Zn Single-Site Curing Activator for a More Sustainable Sulfur Cross-Link Formation in Rubber
Source: Ind Eng Chem Res. 2021 Jul 7;60(28):10180–92. doi: 10.1021/acs.iecr.1c01580 (PMC8411846; doi:10.1021/acs.iecr.1c01580)
Supplement: Supplementary file 1 — ie1c01580_si_001.pdf [file ie1c01580_si_001.pdf]

Supporting information

Design of a Zn single site curing activator for a more sustainable sulfur cross-link formation in rubber

Silvia Mostoni [a], Massimiliano D'Arienzo [a], Barbara Di Credico [a], Lidia Armelao [b,c,d], Marzio Rancan [b], Sandra Dirè [e], Emanuela Callone [e], Raffaella Donetti [f], Antonio Susanna [f], Roberto Scotti *[,a]

[a] Department of Materials Science, INSTM, University of Milano-Bicocca, Via R. Cozzi 55, 20125 Milano, Italy

[b] Institute of Condensed Matter Chemistry and Technologies for Energy, National Research Council of Italy, ICMATE-CNR, via Marzolo 1, 35131 Padua, Italy

[c] Department of Chemical Sciences, University of Padua, Via Marzolo 1, 35131 Padua, Italy

[d] Department of Chemical Sciences and Materials Technologies, National Research Council of Italy, DSCTM-CNR, Piazzale A. Moro 7, 00185 Rome, Italy

[e] "Klaus Müller" Magnetic Resonance Lab., Dept. Industrial Engineering, University of Trento, Via Sommarive 9, 38123 Trento, Italy

[f] Pirelli Tyre SpA, Viale Sarca 222, 20126 Milano, Italy

Corresponding author: Roberto Scotti (email: roberto.scotti@unimib.it)

1. TGA

TGA profiles were registered for both bare SiO_2 and A_X-SiO_2, as reported in Figure S1.

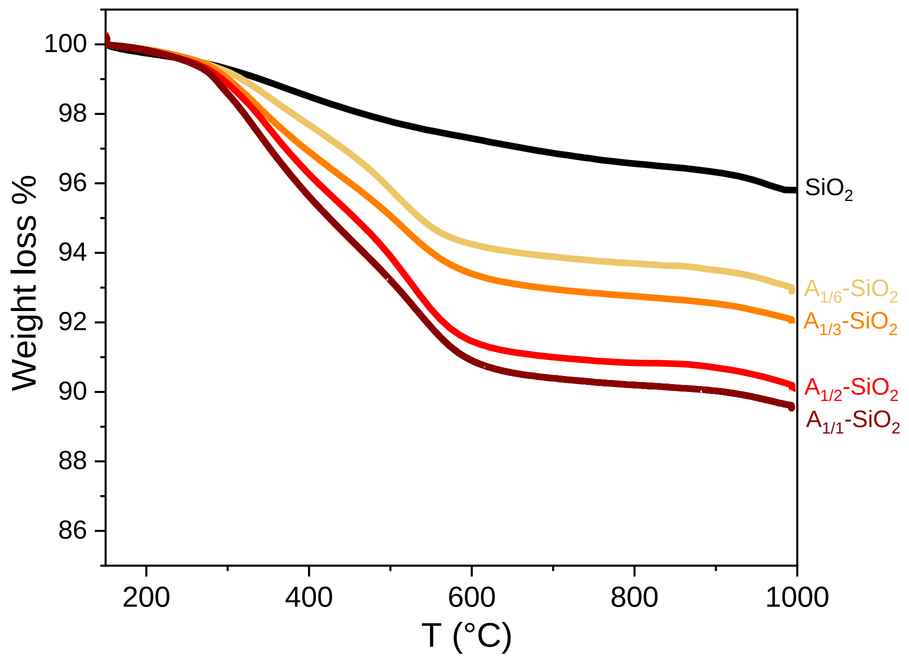

Figure S1. Thermal degradation profiles of A_X-SiO_2 compared to that of bare SiO_2

The weight loss of bare SiO_2 in the range 150-1000°C ($\Delta W_{150\text{-}1000°C}$) was used to estimate the amount of surface silanol groups on silica, considering that water desorption occurs due to the recombination of two -OH groups. For SiO_2, the weight loss could be expressed as follows:

$$\Delta W_{150-1000°C} = \frac{1}{2} \cdot \left(n_{OH} \cdot w_{SiO_2(1000°C)}\right) \cdot MW_{H_2O} \qquad (1)$$

where n_{OH} is the moles of surface OH groups per gram of SiO_2, $w_{SiO2(1000°C)}$ is the weight of SiO_2 measured at 1000°C and MW_{H2O} is the molecular weight of water. From equation 1, n_{OH} was 5.2 mmol g^{-1} for SiO_2.

Considering A_X-SiO_2 samples, $\Delta W_{150\text{-}1000°C}$ was attributed to: i) combustion of the functionalizing groups (-$CH_2CH_2CH_2NH_2$) anchored to SiO_2 NPs after the reaction with APTES; ii) water desorption from the residual surface silanol groups, assuming that each APTES group is bonded to SiO_2 through two covalent bonds and two surface OH groups have been replaced by each APTES bonded to SiO_2 [1]; iii) water desorption from residual hydroxyl groups bonded to APTES units, due to hydrolysis of the third ethoxy group not involved in APTES bonding on the surface. The contributions to the weight loss $\Delta W_{150\text{-}1000°C}$ are reported in the following equation:

$$\Delta W_{150-1000°C} = n_R \cdot MW_R + \frac{1}{2} \cdot \left(n_{OH} \cdot w_{SiO_2(1000°C)} - 2n_R\right) \cdot MW_{H_2O} + \frac{1}{2} \cdot n_{OH-APTES} \cdot MW_{H_2O} \qquad (2)$$

where n_R is the number of APTES moles grafted on SiO_2; MW_R is the molecular weight of (-$CH_2CH_2CH_2NH_2$) groups on SiO_2 (58.0 g mol^{-1}); n_{OH} is 5.2 mmol g^{-1} as determined by TGA on bare SiO_2 sample (equation 1); $n_{OH\text{-}APTES}$ is the number of moles of hydroxyl groups bonded to each APTES unit, that is equal to n_R and the factor ½ is due to the condensation of two hydroxyl groups to generate each water molecule. From equation (2):

$$n_R = \frac{2*\Delta w_{150-1000°C} - n_{OH}*w_{SiO_2(1000°C)}*MW_{H_2O}}{2MW_R - MW_{H_2O}} \qquad (3)$$

From the obtained n_R, the amount of APTES anchored to SiO_2 ($w\%_A$), the reaction yield of SiO_2 functionalization with APTES (Y_A) and the number of APTES molecules anchored onto the SiO_2 surface were calculated:

$$w\%_A = \frac{n_R*MW_R}{m_{SiO2\,(1000°C)}} \times 100 \qquad (4)$$

$$Y_A = \frac{n_R}{n_A} \times 100 \qquad (5)$$

$$\frac{\text{n.molecules}}{\text{surface(nm}^2\text{)}} = \frac{N_A*n_R}{n_{SiO_2}*MW_{SiO_2}*S_{BET}*10^{18}} \qquad (6)$$

where n_A is the nominal moles of APTES used to functionalize SiO_2, N_A the Avogadro number, MW_{SiO2} the molecular weight of SiO_2, S_{BET} the specific surface area of SiO_2 (160 m^2 g^{-1}).

2. SS-NMR

Table S1. Profile fitting results for the 5 components in the ^{29}Si CPMAS NMR spectra of $A_{1/6}$-SiO_2 and $A_{1/2}$-SiO_2 and SiO_2 samples.

Samples	Q^4	Q^3	Q^2	T^3	T^2
**SiO_2**	27.0	62.2	10.9	-	-
**$A_{1/6}$-SiO_2**	33.3	45.4	5.6	8.1	7.6
**$A_{1/2}$-SiO_2**	35.0	31.5	7.4	18.0	8.1

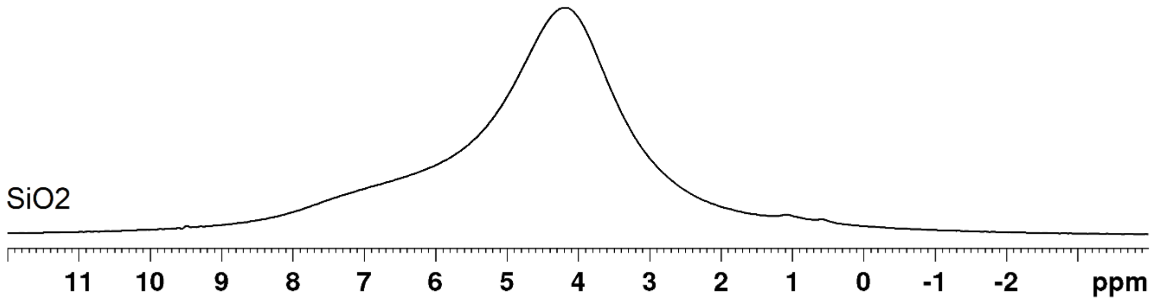

Figure S2. ^{1}H MAS spectrum of SiO_2.

3. EPR

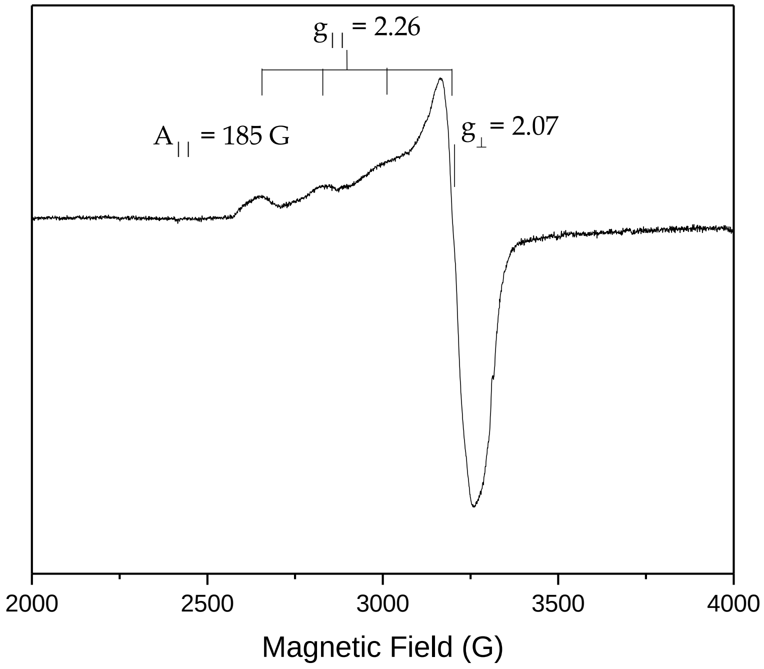

Figure S3. EPR spectrum recorded at 123 K of $Zn_{1/2}A_{1/2}$-SiO_2 doped with Cu (0.01%).

4. TEM of IR NCs

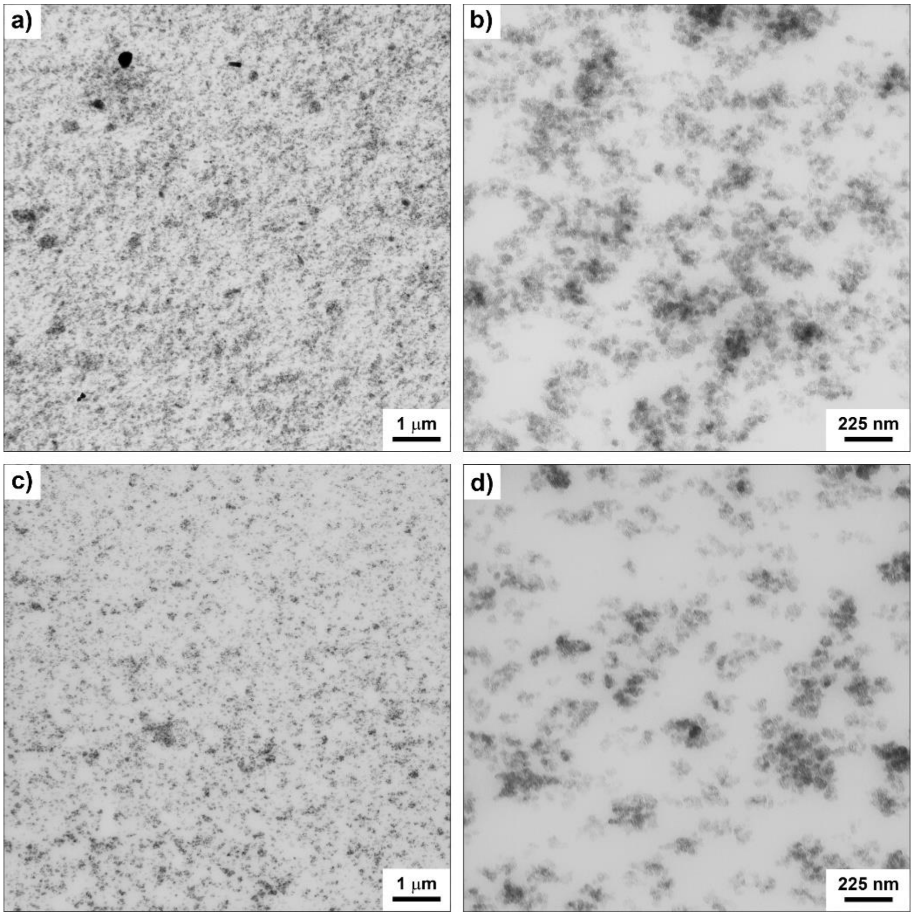

Figure S4. TEM images of (1.5)ZnA-SiO_2/IR (c, d) and (1.5)ZnO-SiO_2/IR (a, b) at two different magnifications.

5. Swelling experiments: Flory-Rehner approach

Swelling experiments were performed to measure the cross-linking densities of ZnA-SiO_2/IR and ZnO-SiO_2/IR. Samples of 20x20x3 mm^3 (m_0 = 1.00 g ± 0.05 g) were immersed in closed vessels filled with 25 mL of toluene at 25°C in the dark to avoid photo-degradation reactions. The samples were swollen for four days, changing the solvent daily with fresh toluene, to eliminate the extracted fractions. At the end, the samples were dried for 24 h at room temperature and the cross-linking densities calculated according to equation (7-8) as reported in the Supporting Information.

The cross-linking densities of ZnA-SiO_2/IR and ZnO-SiO_2/IR were calculated by swelling experiments according to the Flory-Rehner equation. In details, the method makes use of the mass of the rubber samples before (m_0) and after (m_{SW}) the swelling procedure, to calculate the volumetric fraction of the swelled rubber V_R by means of the following equation:

$$V_R = \frac{(m_D - f \cdot m_0) \cdot \rho_p^{-1}}{(m_D - f m_0) \cdot \rho_p^{-1} + m_{SO} \cdot \rho_S^{-1}} \qquad (7)$$

where m_D is the mass of the dried samples after 24 h at room temperature, m_{SO} is the weight of the solvent in the swollen mass ($m_{SO} = m_{SW} - m_D$), $\rho_P = 0.94$ g·cm^{-3} is the IR density, $\rho_s = 0.87$ g·cm^{-3} is the toluene density and f is the fraction of filler NCs as determined by TGA. From this value, it was possible to estimate the cross-linking density ν according to the Flory-Rehner equation[2]:

$$\nu = \frac{[\ln(1 - V_R) + V_R + \chi \cdot V_R^2]}{-2 \cdot \rho_p \cdot V_s \cdot (V_R)^{1/3}} \qquad (8)$$

where $V_S = 105.91$ is the molar volume of toluene and χ is the Flory solvent-polymer interaction term[3], which is 0.43 for toluene-IR[4].

6. Technical NCs

Table S2. Vulcanization parameters of ZnA-SiO_2/IR-T and ZnO-SiO_2/IR-T NCs vulcanized at the two different conditions (30 minutes at 151°C or 10 minutes at 170°C): Mmin = minimum torque; Mmax = maximum torque; t_{S1} = scorch time; t_{90} = time to achieve 90% of M_{max}.

**Sample**	**Vulcanization 30 min, 151°C**				**Vulcanization 10 min, 170°C**			
	**M_{max} [dNm]**	**M_{min} [dNm]**	**t_{S1} [min]**	**t_{90} [min]**	**M_{max} [dNm]**	**M_{min} [dNm]**	**t_{S1} [min]**	**t_{90} [min]**
ZnA-SiO_2/IR-T	40.95	5.95	0.52	7.58	41.3	5.95	0.30	2.19
ZnO-SiO_2/IR-T	28.06	3.30	0.79	11.42	26.96	3.30	0.52	3.29

Table S3. Static and dynamic mechanical properties of ZnA-SiO_2/IR-T and ZnO-SiO_2/IR-T NCs. Static: CA0.1, CA0.5, CA1 and CA3 = tensile modulus at four different elongation level equal to 10%, 50%, 100% and 300%, respectively. CR = Elongation at break. Dynamic: E' = elastic modulus and tanδ = loss values, measured at three different temperatures (10°C, 23°C, 100°C).

**Sample**	**Static Mechanical Properties**					**Dynamic Mechanical Properties**					
						**10°C**		**23°C**		**100°C**	
	**Ca0.1 [MPa]**	**Ca0.5 [MPa]**	**Ca1 [MPa]**	**Ca3 [MPa]**	**CR [MPa]**	**E' [MPa]**	**tanδ**	**E' [MPa]**	**tanδ**	**E' [MPa]**	**tanδ**
ZnA-SiO_2/IR-T	0.61	1.36	2.14	8.77	26.17	6.71	0.22	6.26	0.18	5.56	0.10
ZnO-SiO_2/IR-T	0.54	1.33	2.17	9.39	27.66	6.14	0.16	5.93	0.13	5.45	0.08

7. <u>MCV</u>

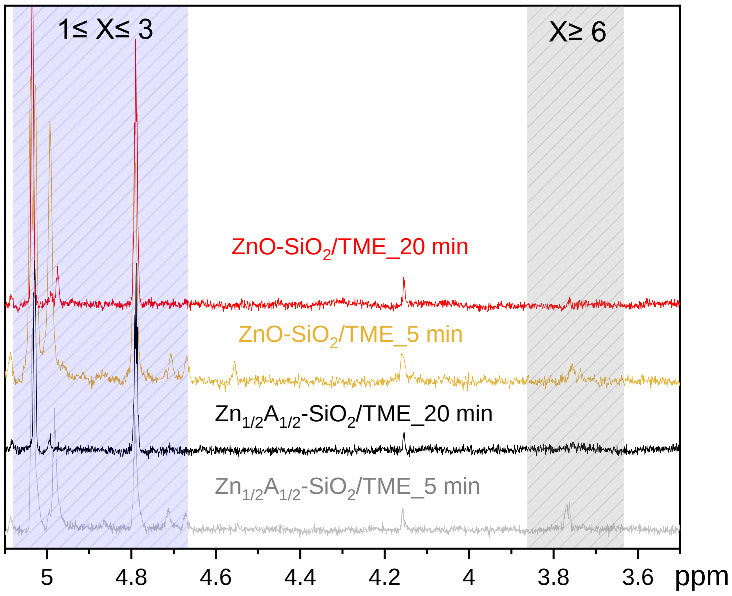

Figure S5. ^{1}H-NMR of $Zn_{1/2}A_{1/2}$-SiO_2/TME and ZnO-SiO_2/TME at reaction times equal to 5 and 20 min.

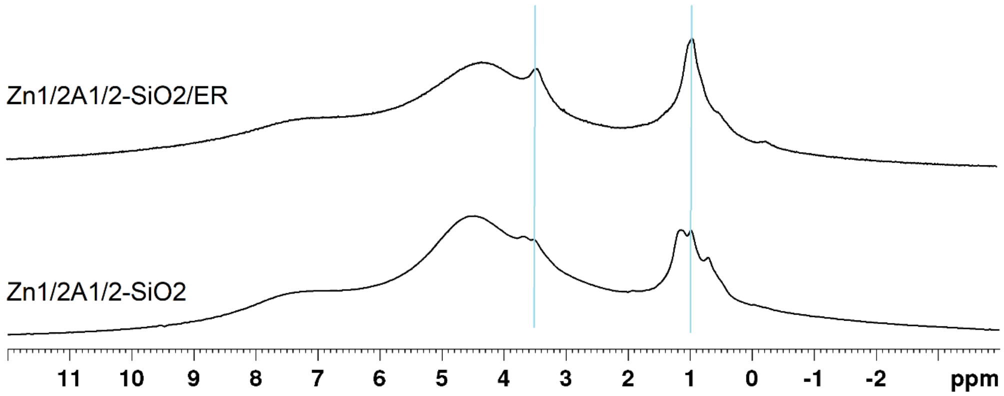

Figure S6. ^{1}H MAS-NMR spectra of $Zn_{1/2}A_{1/2}$-SiO_2/ER compared with that of $Zn_{1/2}A_{1/2}$-SiO_2 before reaction

References

(1) Nigar, H.; Garcia-Banos, B.; Penaranda-Foix, F. L.; Català-Civera, J. M.; Mallada, R.; Santamarìa, J. Amine-Functionalized Mesoporous Silica: A Material Capable of CO2 Adsorption and Fast Regeneration by Microwave Heating. *Am. Inst. Chemcal Eng.* **2016**, *62* (2), 547–555. https://doi.org/10.1002/aic.

(2) Flory, P. J.; Rehner, J. Statistical Mechanics of Cross-Linked Polymer Networks I. Rubberlike Elasticity. *J. Chem. Phys.* **1943**, *11* (11), 512–520.

(3) Flory, P. J. Thermodynamics of High Polymer Solutions. *J. Chem. Phys.* **1942**, *10*, 51–61.

(4) Orwoll, R. A.; Arnold, P. A.; Bucksch, H. Polymer-Solvent Interaction Parameter X. In *Physical Properties of Polymers Handbook*; Mark, J., Ed.; Springer: New York, 2007; pp 233–257. https://doi.org/10.1007/978-3-642-41714-6_31317.
